# High-risk soft-tissue sarcomas in elderly patients: does perioperative radiotherapy improve local control and prognosis?

**DOI:** 10.1186/s12885-026-15629-8

**Published:** 2026-01-31

**Authors:** Tomohiro Fujiwara, Toshiharu Mitsuhashi, Yutaka Nezu, Takashi Tajima, Shinji Miwa, Toshio Kojima, Shuichi Fujiwara, Akira Kawai, Kazuhiro Tanaka, Toshifumi Ozaki

**Affiliations:** 1 Department of Orthopaedic Surgery, Okayama University Graduate School of Medicine, Dentistry, and Pharmaceutical Sciences, Okayama, Japan; 2 Centre for Innovative Clinical Medicine, Medical Development Field, Okayama University, Okayama, Japan; 3 Department of Orthopaedic Surgery, Yokohama City University, Kanagawa, Japan; 4 Department of Orthopaedic Surgery, Kyorin University Faculty of Medicine, Tokyo, Japan; 5 Department of Orthopaedic Surgery, Graduate School of Medical Sciences, Kanazawa University, Kanazawa, Japan; 6 Department of Orthopaedic Surgery, Nihon University School of Medicine, Tokyo, Japan; 7 Department of Orthopaedic Surgery, Kobe University Graduate School of Medicine, Hyogo, Japan; 8 Department of Musculoskeletal Oncology, National Cancer Centre Hospital, Tokyo, Japan; 9 Department of Advanced Medical Sciences, Oita University Faculty of Medicine, Oita, Japan

**Keywords:** Soft-tissue sarcoma, High-risk, Surgery, Perioperative radiotherapy, Elderly patients

## Abstract

**Aims:**

Accumulating evidence suggests that advanced age is associated with poor local control and prognosis in patients with soft-tissue sarcomas (STSs), highlighting the need to optimise treatment for this age group. However, real-world data on treatment details and outcomes in elderly patients are limited. This study aimed to clarify the role of perioperative radiotherapy (RT) for treating high-risk STSs in elderly patients.

**Methods:**

Patients aged ≥ 70 years who underwent surgery for localised, high-grade, deep-seated non-small round cell STSs measuring ≥ 5 cm were included in the Bone and Soft Tissue Tumour Registry in Japan. Patients with small-round cell STSs or myxoid liposarcomas, or those who received perioperative chemotherapy or intraoperative RT, were excluded.

**Results:**

Among the 1,214 patients who met the criteria, 47 (4%), 219 (18%), and 2 (0.2%) received neoadjuvant, adjuvant, and both neoadjuvant and adjuvant RT, respectively. The 5- and 10-year disease-specific survival (DSS) rates were 72.7% and 64.7%, respectively. Tumour size ≥ 10 cm, intralesional margin, and local recurrence were associated with poorer DSS; however, perioperative RT did not affect DSS. The 5- and 10-year cumulative probabilities of local recurrence (LR) were 14.6% and 19.5%, respectively. Trunk wall tumours, dedifferentiated liposarcomas, marginal margins, and intralesional margins were associated with a higher probability of LR. Adjuvant RT was associated with a reduced LR probability in patients with intralesional (*p* = 0.005) or marginal margins (*p* = 0.044); however, no such benefit was observed in patients with wide margins, who constituted the majority of the cohort, resulting in no significant association between perioperative RT and LR in overall analyses. In the propensity score-matched cohort, no significant differences in DSS or cumulative probability of LR were observed between patients with and without perioperative RT.

**Conclusion:**

Adjuvant RT was associated with reduced LR rates in elderly patients with high-risk STSs who had intralesional or marginal margins. However, because most patients achieved wide margins and no benefit of perioperative RT was observed in this group, RT was not associated with reduced LR or improved survival in the overall or propensity score–matched analyses. Prospective trials are warranted to define the role of perioperative RT in elderly patients with high-risk STSs.

**Supplementary Information:**

The online version contains supplementary material available at 10.1186/s12885-026-15629-8.

## Introduction

Soft-tissue sarcomas (STSs) are a diverse group of malignant mesenchymal tumours with > 50 histological entities [1–3]. STSs occur in < 1% of all malignancies, and according to the American Cancer Society, approximately 13,000 cases are newly diagnosed annually in the USA [4]. The Soft Tissue Tumour Registry by the Japanese Orttely 1,800 cases had been registered in Japan in 2020 [5]. The mainstay of treatment for localised STSs is surgical resection. The standard surgical procedure involves a wide excision with negative margins [1, 3, 6–8]. Neoadjuvant and/or adjuvant chemotherapy is considered for high-grade STSs measuring > 5 cm that are located deeper than the investing fascia in the extremities and trunk [9]. Neoadjuvant and/or adjuvant radiotherapy (RT) is considered for high-grade deep STSs measuring > 5 cm, particularly when wide margins are unlikely to be achieved or resection margins are inadequate, typically from intralesional to marginal margins [9]. However, multidisciplinary treatment is sometimes challenging to implement in elderly patients due to their vulnerability.

STSs in elderly people are characterized by unique patterns of histological subtypes compared with those in younger patients. Undifferentiated pleomorphic sarcoma (UPS), which was previously described as a malignant fibrous histiocytoma, and myxofibrosarcoma (MFS) are more prevalent [10, 11]. These subtypes are characterized by frequent radiological and pathological observation of infiltrative growth patterns [12]. Importantly, Biau et al. demonstrated that elderly patients presented with larger and higher-grade tumours, and the proportion of positive margins at resection increased progressively with age. The proportions of positive margins were 13%, 15.3%, 20%, 21.9%, and 27.2% in patients aged < 30, 20–44, 45–59, 60–74, and ≥ 75 years, respectively (*p* < 0.001), which might be associated with the increased proportions of these infiltrative STS subtypes [10]. They concluded that advanced age was a negative factor that influenced local control and survival [10]. These backgrounds suggest the expanding role of adjuvant RT, particularly in elderly patients. However, real-world data on the use and efficacy of adjuvant RT in elderly patients with STSs are needed.

Based on the above background data, this study aimed to clarify the role of neoadjuvant (preoperative)/adjuvant (postoperative) RT in high-risk (high-grade, deep-seated, ≥ 5 cm), non-round cell STSs in elderly patients. This study aimed to address the following research questions: 1) What are the proportions of elderly patients with high-risk STS who receive perioperative RT? 2) What is the effect of perioperative RT on local control? 3) What is the effect of perioperative RT on survival? Accordingly, retrospective analyses were conducted using the Japanese Nationwide Bone and Soft Tissue Tumour Registry (BSTTR) database.

## Patients and methods

### Data source

The data source was the BSTTR, which is a nationwide registry for bone and soft-tissue tumours, based at the National Cancer Centre Hospital and funded by the Japanese Orthopaedic Association (JOA). Data were collected from 89 JOA-certified hospitals in which registration is mandatory, and from other hospitals in which data registration is voluntary. All data, including tumour- and treatment-related data, and oncological outcomes are registered by the physicians, and updated annually. This study was approved by the Institutional Review Board of the Okayama University Hospital (2407–031). The need for informed consent was waived by the committee due to the retrospective nature of the study and the use of anonymised data. An opt-out option was permitted for patients who had the opportunity to decline study participation.

**Fig. 1:**
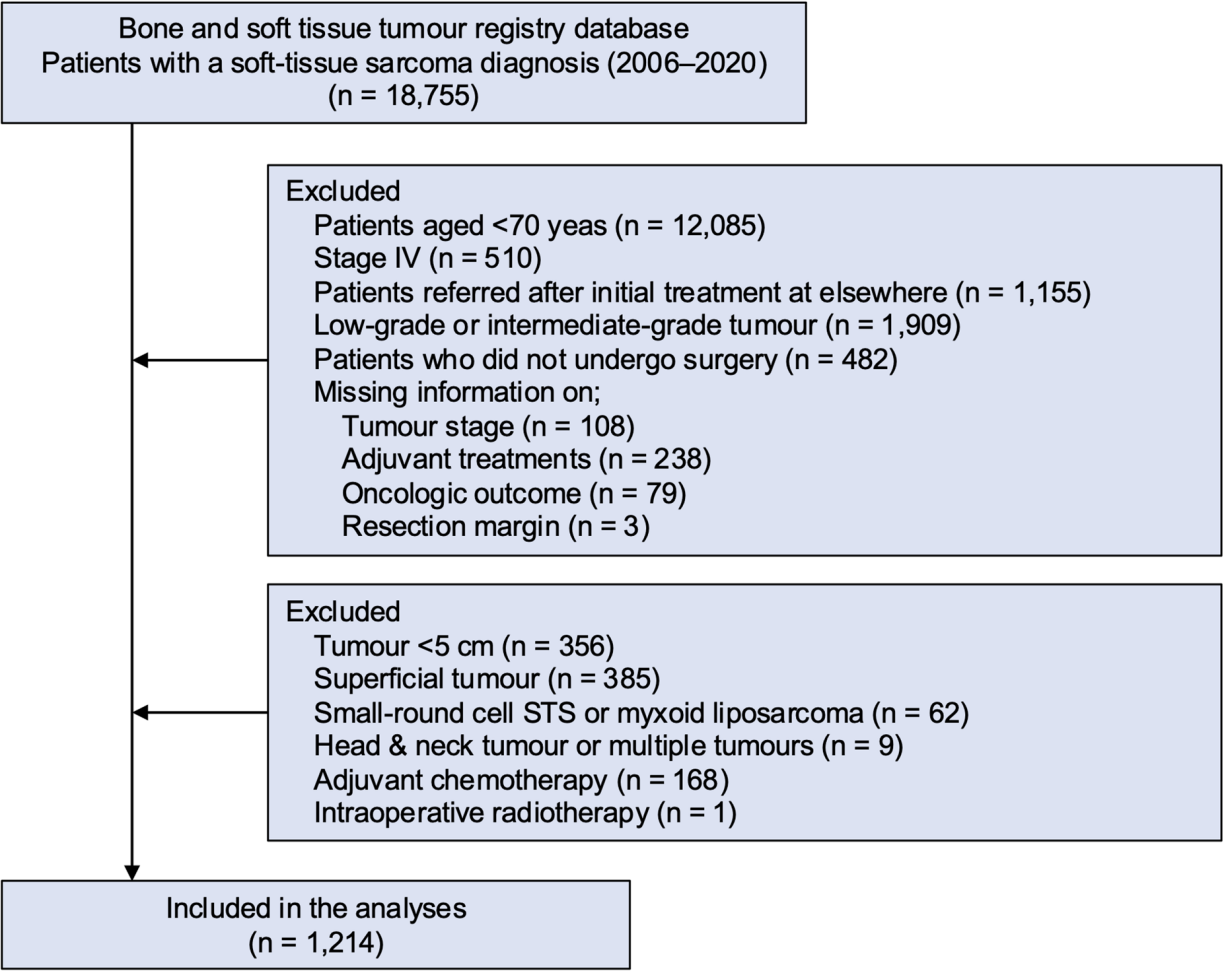
Flow diagram of the study inclusion and exclusion

Data registered from 2006 to 2020 were obtained from the BSTTR. The inclusion criteria were elderly patients aged ≥ 70 years who underwent surgical excision for a high-grade, localised, non-small round cell STS. Of the 18,755 patients who were registered to the BSTTR, those who were < 70 years old (*n* = 12,085), had stage IV tumours at diagnosis (*n* = 510), had been referred to the treating hospital after initial treatments elsewhere (*n* = 1,155), had low- or intermediate-grade STSs (*n* = 1,909), had no surgical treatments (*n* = 482), and had missing information about tumour stage (*n* = 108), adjuvant treatments (*n* = 238), oncological event (*n* = 79), and resection margin (*n* = 3) were excluded. Among 2,186 patients left, those who had tumours measuring < 5 cm (*n* = 356), superficial tumours (*n* = 385), small-round cell STSs or myxoid liposarcomas (*n* = 62), underwent neoadjuvant/adjuvant chemotherapy (*n* = 168), and received intraoperative RT (*n* = 1) were excluded from the analyses because these are potential biases in evaluating the efficacy of neoadjuvant/adjuvant RT for high-risk STSs (Fig. 1).

The following data were extracted from the BSTTR: basic demographic details (age, sex, status at the first visit such as newly diagnosed or referred after initial treatment elsewhere, and date of referral), tumour-related information (diagnosis date, diagnosis method [pathology or clinical], tumour grade, site, and depth, and metastases at the time of diagnosis), treatment-related information (surgery for the primary tumour, use and purpose of chemotherapy and/or RT, and sequence of chemotherapy and RT), and oncological outcomes at the final review. Surgical margins were recorded as radical, wide, marginal, or intralesional margins using the system established by Enneking et al. [13]. Radical or wide margins correspond to R0 (no residual tumour), intralesional margins correspond to R1 or R2 resections (microscopic or macroscopic residual tumour), and marginal margins generally correspond to R1 (microscopic residual tumour), although in some cases they may also correspond to R0, depending on the pathological assessment [14].

### Statistical analysis

The primary study outcome was disease-specific survival (DSS), whereas the secondary outcome was cumulative probability of local recurrence (LR). The Kaplan–Meier method was employed to determine DSS, with time zero defined as the diagnosis date and censored at the date of the last follow-up. The disease-specific death was determined in each participating institution based on clinician adjudication or on death certificates and then registered in the BSTTR. Univariate analysis was conducted by comparing the groups using the log-rank test and factors that are biologically considered as significant and without high multicollinearity were incorporated into a multivariable analysis. The cause-specific Cox proportional hazards model was used to compute hazard ratios (HRs) and their 95% confidence intervals (CIs). Cancer-related deaths were defined as the event of interest, whereas deaths from other causes and patients alive at last follow-up were treated as censored observations. Before estimating the Cox regression model, the proportional hazards assumption was tested for each variable using the time-varying Cox model, failing to reject the null hypothesis of proportional hazards when the *p*-value was > 0.05. The cumulative incidence of LR was analysed using a competing risk framework. Death from any cause was regarded as a competing event. In the univariable analyses, groups were compared using the Gray’s test; in multivariable analyses, a Fine-Gray subdistribution hazard model was used. The variables of each group were compared using the chi-square test or Fisher’s exact test. A *p*-value ≤ 0.05 was considered significant. Logistic regression was applied to adjust for bias, including the following variables for the propensity score calculation: age, sex, tumour site, tumour size, diagnosis, and surgical margin. Using calipers (0.2) of the SD of the log of the propensity score, a propensity score analysis with 1:1 matching was conducted without matching replacement. Covariate balance was evaluated using standardized mean differences (SMDs) and variance ratios, with SMD values below 0.1 and variance ratios between 4/5 and 5/4 generally considered acceptable thresholds for covariate balance [15, 16]. As a supplementary analysis, stabilised inverse probability of treatment weighting (sIPTW) was applied. For patients receiving adjuvant RT, weights were defined as the marginal probability of treatment divided by the propensity score; for those not receiving RT, as the marginal probability of no treatment divided by one minus the propensity score. After adjustment for potential confounders via propensity score matching and sIPTW, Kaplan–Meier survival curves were created. Statistical analyses were conducted using the R statistics (version 4.3.3) and Stata 19.5/MP (StataCorp, College Station, TX, USA).

## Results

### Characteristics of patients who received neoadjuvant/adjuvant RT

This study enrolled 1,205 patients with localised, high-grade STSs of the extremity or trunk wall. The median age was 78 (interquartile range [IQR], 74–83) years, and 726 (60%) patients were male. The primary tumour sites were the lower extremity in 687 (57%) patients, the trunk wall in 363 (30%), and the upper extremity in 155 (13%). The median tumour size was 10.0 cm (range, 5.0–12.0 cm). The histological diagnoses were UPS (*n* = 461; 38%), myxofibrosarcoma (*n* = 266; 22%), dedifferentiated liposarcoma (*n* = 194; 16%), and others (*n* = 168; 14%; Table 1). The surgical margins were wide in 975 (81%) patients, marginal in 159 (13%), and intralesional in 71 (6%).

**Table 1:** Patient characteristics according to the RT sequence

RT sequence	Total		No RT		Neoadjuvant RT		Adjuvant RT		Neoadjuvant + adjuvant RT		*p* value
	(*n* = 1,205)		(*n* = 941; 78%)		(*n* = 46; 4%)		(*n* = 216; 18%)		(*n* = 2: 0.2%)		
	N	% (range)	N	%	N	%	N	%	N	%	
Age											0.097
< 80 years	688	57%	526	76%	31	5%	131	19%	0	0%	
≥ 80 years	517	43%	415	80%	15	3%	85	16%	2	0.4%	
Median (years)	78	(70–98)	78	(70–97)	75	(70–91)	77	(70–98)	87	(82–92)	
Sex											0.222
Male	726	60%	576	79%	26	4%	124	17%	0	0%	
Female	479	40%	365	76%	20	4%	92	19%	2	0.4%	
Tumour site											0.018
Lower extremity	687	57%	552	80%	23	3%	111	16%	1	0.1%	
Upper extremity	155	13%	104	67%	7	5%	44	28%	0	0%	
Trunk	363	30%	285	79%	16	4%	61	17%	1	0.3%	
Size											0.033
< 10 cm	589	49%	479	81%	21	4%	89	15%	0	0%	
≥ 10 cm	616	51%	462	75%	25	4%	127	21%	2	0.3%	
Diagnosis											0.216
UPS	461	38%	364	79%	20	4%	76	16%	1	0.2%	
Myxofibrosarcoma	266	22%	192	72%	15	6%	59	22%	0	0%	
Dedifferentiated LPS	194	16%	162	84%	4	2%	28	14%	0	0%	
Leiomyosarcoma	116	10%	91	78%	2	2%	23	20%	0	0%	
Others	168	14%	132	79%	5	3%	30	18%	1	0.6%	
Margin											< 0.001
Intralesional	71	6%	34	48%	0	0%	37	52%	0	0%	
Marginal	159	13%	86	54%	8	5%	65	41%	0	0%	
Wide	975	81%	821	84%	38	4%	114	12%	2	0.2%	
LR											0.446
No	1042	86%	819	79%	41	4%	180	17%	2	0.2%	
Yes	163	14%	122	75%	5	3%	36	22%	0	0%	
Year											0.152
–2010	235	20%	182	77%	8	3%	44	19%	1	0.4%	
2011–2015	410	34%	328	80%	8	2%	74	18%	0	0%	
2016–2020	560	46%	431	77%	30	5%	98	18%	1	0.2%	

Of the 1,205 patients included, 46 (4%), 216 (18%), and 2 (0.2%) received neoadjuvant RT, adjuvant RT, and both neoadjuvant and adjuvant RT, respectively. The tumour site (*p* = 0.018), tumour size (*p* = 0.033), and surgical margin (*p* < 0.001) were associated with the RT sequence (Table 1). Overall, perioperative RT was administered to patients with larger tumours (*p* = 0.005; Supplementary Table 1) and inadequate (intralesional and marginal) resection margins (*p* < 0.001; Supplementary Table 1). Regarding the RT sequence, approximately half of the patients who achieved intralesional (*n* = 37; 52%) or marginal (*n* = 65; 41%) received adjuvant RT (Table 1).

### The role of neoadjuvant/adjuvant RT in DSS

During the last follow-up, 245 (20%) patients had died of the disease. The 5- and 10-year DSS rates were 72.9% and 64.8%, respectively (Supplementary Fig. 1). In the univariable analyses, tumour size (*p* < 0.001), histological diagnosis (*p* = 0.015), surgical margin (*p* = 0.003), and LR (*p* < 0.001) were significantly associated with DSS (Supplementary Fig. 2, Supplementary Table 2). However, adjuvant RT did not significantly affect DSS (5-year DSS; RT +, 69.8%; RT −, 73.9%; *p* = 0.221; Supplementary Fig. 1, Supplementary Table 2). This finding was confirmed in the subgroup analysis according to histological subtypes. In the multivariable analyses, a tumour measuring ≥ 10 cm (HR = 1.53; 95% CI, 1.17–1.99; versus 5–10 cm, reference), intralesional margin (HR = 1.67; 95% CI, 1.07–2.61; versus wide margin, reference), and LR (HR = 2.20; 95% CI, 1.63–2.98; versus no LR, reference) were independently associated with poorer DSS (Supplementary Table 2). Therefore, DSS was significantly associated with the local control of high-risk STSs in elderly patients.

### Role of neoadjuvant/adjuvant RT in local control

**Fig. 2:**
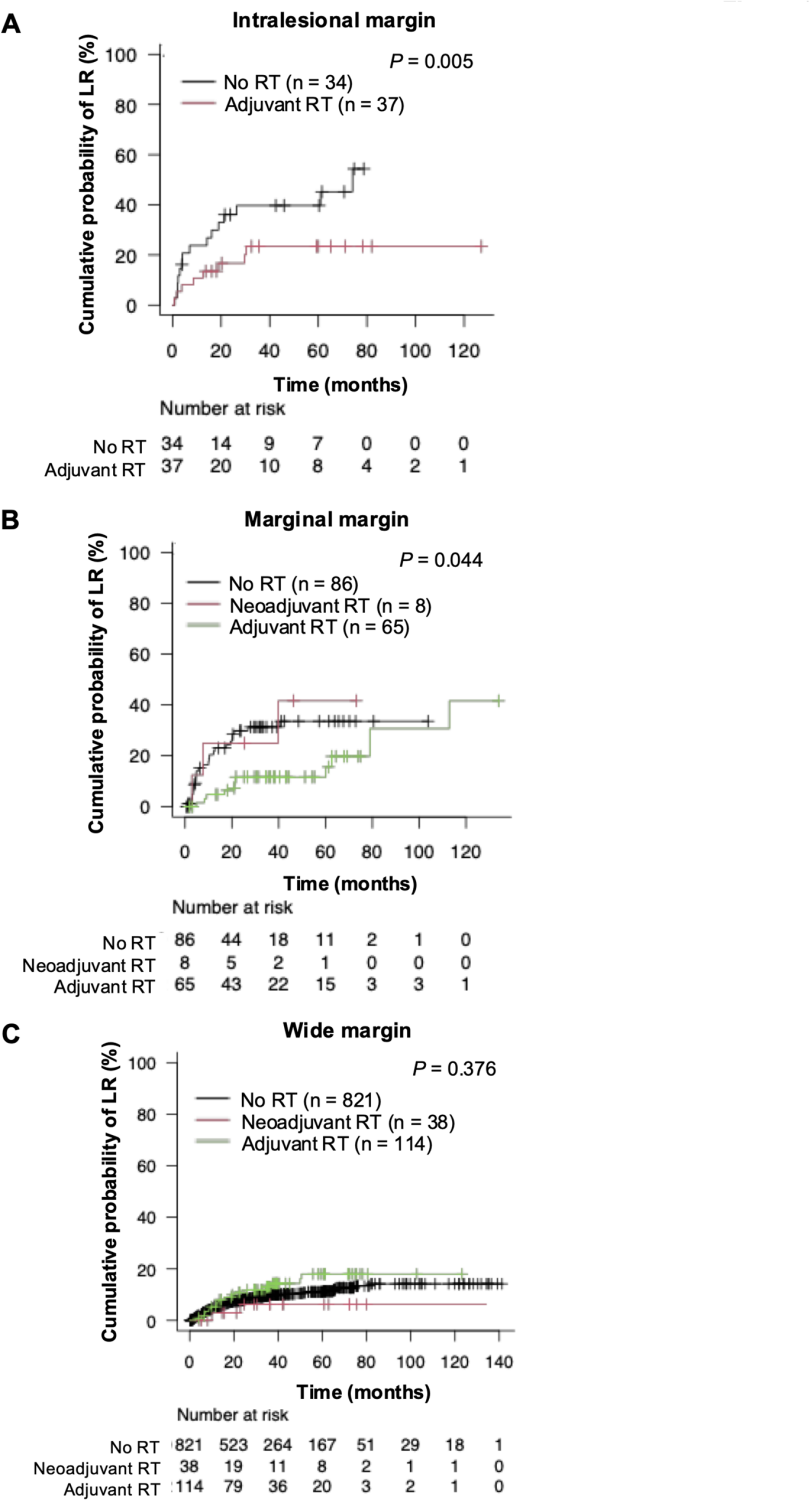
Cumulative probability of local recurrence stratified by the administration of adjuvant RT in patients with (**A**) intralesional (Gray’s test,* P*= 0.005), (**B**) marginal (Gray’s test,* P*= 0.044), and (**C**) wide (Gray’s test,* P*= 0.376) margins

At the last follow-up, LR was noted in 163 (13.5%) patients. The crude cumulative probability of LR at 5 and 10 years were 14.6% and 19.5%, respectively (Supplementary Fig. 3). In the univariable analyses, tumour size (*p* = 0.015), tumour site (*p* < 0.001), histological diagnosis (*p* < 0.001), and resection margin (*p* < 0.001) were significantly associated with LR (Supplementary Fig. 4). However, perioperativeRT did not significantly affect cumulative probability of LR (5-year; RT +, 16.3%; RT −, 14.1%; *p* = 0.506; Supplementary Fig. 3). In the multivariable analyses, trunk (HR = 1.82; 95% CI, 1.32–2.51; versus lower extremity, reference), dedifferentiated liposarcoma (HR = 1.81; 95% CI, 1.21–2.71; versus UPS, reference), marginal margin (HR = 2.28; 95% CI, 1.51–3.38; versus wide margin, reference), and intralesional margin (HR = 3.27; 95% CI, 1.02–5.28; versus wide margin, reference) were independently associated with poorer local control (Supplementary Table 3). Of note, adjuvant RT was significantly associated with the reduced probability of LR in patients with intralesional (*p* = 0.005) or marginal (*p* = 0.044) margins (Fig. 2a, b). However, this association was not observed in patients with wide margins (*p* = 0.376; Fig. 2c), who represented most of the cohort; consequently, no significant effect of perioperative RT on local control was observed in the overall analysis.

### Propensity score analyses

**Fig. 3:**
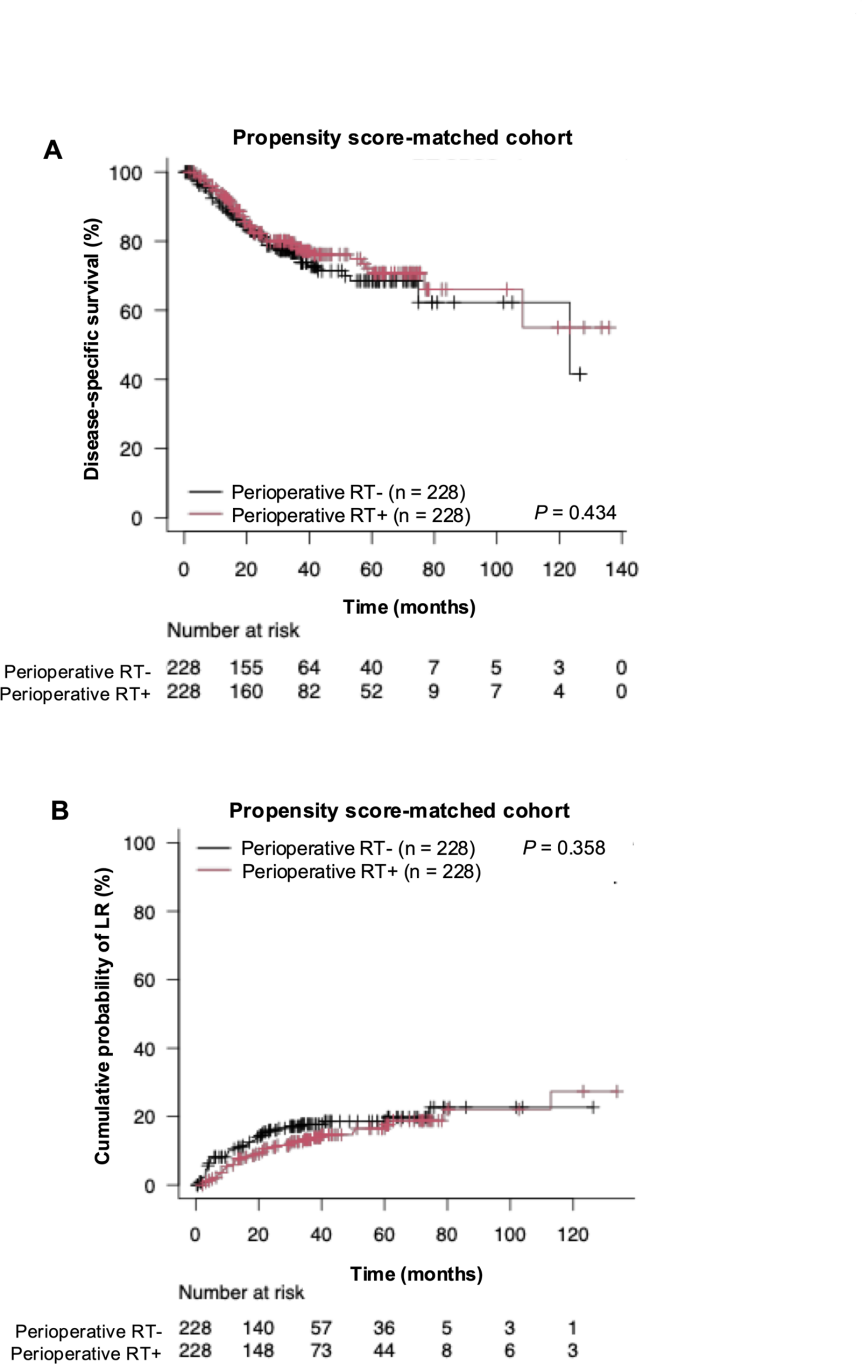
Kaplan–Meier curve showing (**A**) disease-specific survival (log-rank test,* P* = 0.434) and (**B**) cumulative probability of local recurrence (Gray’s test,* P* = 0.358) after propensity score matching

After propensity score matching, a new cohort of 456 patients with and without perioperative RT was created with appropriately balanced baseline covariates. No difference in patient characteristics was found between the two groups (Supplementary Table 4–6). In this cohort, no significant difference in DSS was noted, and the 5-year DSS rates were 72.1% and 68.5% in patients with and without perioperative RT, respectively (*p* = 0.434; Fig. 3 and Supplementary Fig. 5). Similarly, no significant differences in cumulative probability of LR were found, with 16.5% and 18.6% at 5 years in patients with and without perioperative RT, respectively (*p* = 0.358; Fig. 3).

## Discussion

In this study that used a nationwide database, approximately 22% (neoadjuvant, 4%; adjuvant, 18%; both neoadjuvant and adjuvant, 0.2%) of the patients received perioperative RT. Perioperative RT appears to be used in a smaller population in Japan than in Europe or the USA; however, the success rates in local control are nearly identical. Hoven-Gondorie et al. reviewed the treatment modalities and prognoses of patients according to the age group in a patient cohort in the Netherlands and revealed that perioperative RT was administered in 47%, 40%, 36%, and 27% of patients aged 70–74, 75–79, 80–84, and 85 +, respectively [17]. Al-Refaie et al. analyzed the data of elderly patients registered in the Surveillance Epidemiology and End Results registries (SEER) database in the USA, which demonstrated that perioperative RT to limb-sparing surgery was administered in 64% (preoperative RT, 10.3%; postoperative RT, 54.1%), 53% (preoperative RT, 7.4%; postoperative RT, 45.8%), 54% (preoperative RT, 8.4%; postoperative RT, 45.3%), and 41% (preoperative RT, 3.7%; postoperative RT, 37.4%) patients aged 70–74, 75–79, 80–84, and 85 +, respectively [18]. These differences in the rate of perioperative RT administration among countries might be attributable to differences in the consensus and clinical practice guidelines. The European Society for Medical Oncology guidelines state that the typical wide excision followed by RT is a standard treatment for high-grade (grade 2–3) deep STSs (evidence level II, recommendation grade C) measuring > 5 cm [19]. In the Japanese clinical practice guidelines, perioperative RT is not regarded as a standard treatment modality but is suggested with careful consideration of the survival benefits and risk of complications (evidence level C, recommendation grade 2) [19].

In several studies, perioperative RT was found to be effective for local control and preserves the function of the affected extremity [20, 21]. In a prospective, randomised study by the National Cancer Institute in the USA, Rosenberg et al. reported that the LR rate was 15% in 27 patients with high-grade STSs in the extremities treated with limb-sparing surgery followed by 60–70 Gy of RT, whereas none in 16 patients who underwent amputation [22]. A long-term observational study confirmed a reduced LR rate after the addition of adjuvant RT [23, 24]. However, in the present study, local control was comparable between the RT + and RT − groups in the entire cohort, although the cumulative probability of LR was significantly reduced in the RT + group of patients with marginal or intralesional margins. This finding is likely attributable to the absence of a beneficial effect of perioperative RT in patients with wide margins, who constituted the majority of the cohort. The association between adjuvant RT and improved local control suggested by the margin-positive subgroup analyses was not evident after propensity score matching, indicating that the observed benefit may be attributable, at least in part, to treatment-selection mechanisms and residual confounding inherent to observational data. Our findings regarding adjuvant RT in patients with insufficient margins were consistent with previous studies: Alektiar et al. reported that, in patients with extremity STSs, adjuvant RT after R1 resection resulted in improved local control [25]. Regarding the RT sequence, no significant difference was observed between the neoadjuvant RT and adjuvant RT groups, consistent with the results of a published study [26]. In general, the total RT dose is lower and the irradiated volume is smaller in the neoadjuvant setting than in the adjuvant setting, which may result in a lower incidence of fibrosis and edema with neoadjuvant RT [9]. Because this retrospective cohort study was limited by selection bias, a prospective clinical trial is necessary to further evaluate the role of perioperative RT for high-grade STSs in elderly patients.

Previous studies have reported controversial results regarding the role of perioperative RT in survival outcomes [9, 27]. In a prospective, randomised study conducted at the US National Cancer Institute, no significant difference in 5-year survival was observed between patients who underwent limb-salvage surgery followed by adjuvant RT and those who were amputated; the 5-year survival rates were 83% and 88% in the limb-salvage surgery + adjuvant RT and amputation groups, respectively (*p* = 0.99) [22]. A long-term follow-up study of a randomised, prospective trial of limb-sparing surgery with and without adjuvant RT confirmed no statistical difference in survival outcomes; the 10- and 20-year survival rates were 77% and 64% for patients who underwent surgery alone and 82% and 71% for those who underwent surgery followed by adjuvant RT, respectively (*p* = 0.22) [23]. In a retrospective study of the SEER database, Koshy et al. reported that RT was associated with improved survival in patients with high-grade STSs who underwent limb-sparing surgery; the 3-year overall survival rates were 73% and 63% in the RT + and RT − group, respectively (*p* < 0.001) [28]. This study demonstrated the lack of survival benefit of perioperative RT, which was confirmed in the propensity-matched cohort. However, the margin status and LR independently affected survival outcomes in the entire cohort, indicating the possibility of improved survival outcomes if local control is further improved. These are most likely attributable to the under-powered sample size and selection bias. To evaluate the efficacy of perioperative RT for survival without selection bias, a prospective randomised controlled trial involving a larger number of patients would be required.

This study has several limitations. First, this study has a retrospective design and inherent selection biases. Second, analyses were performed using a national database with information gathered from ≥ 89 hospitals; therefore, the treatment policy may vary among hospitals, and information about the reasons for selecting each treatment for each patient was unavailable. Third, the database does not contain detailed RT information, such as dose, technique, and fractionation. In addition, data on RT-related complications are unavailable. Therefore, the efficacy and safety of RT could not be extensively evaluated based on these data. Fourth, we acknowledge the possibility of misclassification bias in determining disease-specific death across participating institutions. Fifth, data regarding comorbidities, performance status, frailty or postoperative complications, all of which influence RT indication in elderly patients, were unavailable from the BSTTR. Sixth, information regarding whether a marginal margin corresponds to R0 or R1 are unavailable from the BSTTR database. In addition, even assuming optimal allocation of perioperative RT, the relatively small absolute number of patients with intralesional resections may have limited the statistical power to detect a survival difference in this subgroup. Finally, indications for perioperative RT, such as proximity of the tumour to the major neurovascular bundles or vital organs, were not available in the database. Perioperative RT might be administered to tumours at high risk of LR because of their close proximity to vital structures. Detailed information on anatomic site distribution in terms of proximity to the major vascular bundles or bones and surgeon or hospital volume, which potentially influence the intralesional margin rate or RT indication, were unavailable from the BSTTR database. Further analyses with larger cohorts and longer follow-up periods are required to reach clearer conclusions on the oncological significance of perioperative RT in the management of STSs in elderly populations.

In summary, this study elucidated the role of perioperative RT in elderly patients aged ≥ 70 years who underwent surgical excision for localised, high-risk (high-grade, deep-seated, and ≥ 5 cm) STSs. Local control was significantly associated with survival outcomes. Adjuvant RT was significantly associated with a reduced probability of LR in patients with intralesional or marginal margins. However, no such benefit was observed in patients with wide margins, who comprised the majority of the cohort. Consequently, perioperative RT was not associated with improved local control or survival in the overall analyses, nor after propensity score matching. No significant differences in DSS or the cumulative probability of LR were observed between neoadjuvant and adjuvant RT. These data support a treatment strategy of administering perioperative RT to high-risk STSs with close or contaminated surgical margins, regardless of histological diagnosis. Considering the selection bias inherent to observational data, a prospective, randomised controlled trial can be considered to further evaluate the efficacy of perioperative RT for localised, high-risk STSs in this age group.

## Supplementary Information

Supplementary Material 1.

## Data Availability

The data that support the findings of this study are available from the Japanese Orthopedic Association committee, but restrictions apply to the availability of these data, which were used under license for the current study, and so are not publicly available. Data are however available from the authors upon reasonable request and with permission of the Japanese Orthopedic Association committee.

## References

[1] Clark MA, Fisher C, Judson I, Thomas JM. Soft-tissue sarcomas in adults. N Engl J Med. 2005;353(7):701–11. 16107623 10.1056/NEJMra041866

[2] Christopher D, Fletcher JA, Bridge P. WHO classification of tumours of soft tissue and bone. International agency for research on cancer 4th edition Lyon. 2013:110–111.

[3] Casali PG, Abecassis N, Aro HT, Bauer S, Biagini R, Bielack S, et al. Soft tissue and visceral sarcomas: ESMO-EURACAN Clinical Practice Guidelines for diagnosis, treatment and follow-up. Ann Oncol. 2018;29(Suppl 4):iv51–67. 29846498 10.1093/annonc/mdy096

[4] American Cancer Society. Soft tissue sarcoma. 2024. https://www.cancerorg/cancer/types/soft-tissue-sarcoma/about/key-statisticshtml.

[5] Japanese Orthopaedic Association Musculoskeletal Tumor Committee. Soft Tissue Tumor Registry in Japan. National Cancer Center; Tokyo; 2020.

[6] Dangoor A, Seddon B, Gerrand C, Grimer R, Whelan J, Judson I. UK guidelines for the management of soft tissue sarcomas. Clin Sarcoma Res. 2016;6:20. 27891213 10.1186/s13569-016-0060-4 PMC5109663

[7] Fujiwara T, Grimer RJ, Evans S, Medellin Rincon MR, Tsuda Y, Le Nail L-R, et al. Impact of NICE guidelines on the survival of patients with soft-tissue sarcomas. Bone Joint J. 2021;103(3):569–77. 33641420 10.1302/0301-620X.103B3.BJJ-2020-0743.R1

[8] Grimer R, Judson I, Peake D, Seddon B. Guidelines for the management of soft tissue sarcomas. Sarcoma. 2010;2010:506182. 20634933 10.1155/2010/506182 PMC2903951

[9] Kawai A, Araki N, Ae K, Akiyama T, Ozaki T, Kawano H, et al. Japanese Orthopaedic Association (JOA) clinical practice guidelines on the management of soft tissue tumors 2020-secondary publication. J Orthop Sci. 2022;27(3):533–50. 35339316 10.1016/j.jos.2021.11.023

[10] Biau DJ, Ferguson PC, Turcotte RE, Chung P, Isler MH, Riad S, et al. Adverse effect of older age on the recurrence of soft tissue sarcoma of the extremities and trunk. J Clin Oncol. 2011;29(30):4029–35. 21931025 10.1200/JCO.2010.34.0711

[11] Okamoto M, Kito M, Yoshimura Y, Aoki K, Suzuki S, Tanaka A, et al. The status quo of treatment and clinical outcomes for patients over 80 years of age with high-grade soft tissue sarcoma: report from the soft tissue tumor registry in Japan. Jpn J Clin Oncol. 2018;48(10):900–5. 30137471 10.1093/jjco/hyy118

[12] Imanishi J, Slavin J, Pianta M, Jackett L, Ngan SY, Tanaka T, et al. Tail of superficial myxofibrosarcoma and undifferentiated pleomorphic sarcoma after preoperative radiotherapy. Anticancer Res. 2016;36(5):2339–44. 27127141

[13] Enneking WF, Spanier SS, Goodman MA. A system for the surgical staging of musculoskeletal sarcoma. Clin Orthop Relat Res. 1980;153:106–20. 7449206

[14] Endo M, Lin PP. Surgical margins in the management of extremity soft tissue sarcoma. Chin Clin Oncol. 2018;7(4):37. 30173528 10.21037/cco.2018.08.10

[15] Ali MS, Prieto-Alhambra D, Lopes LC, Ramos D, Bispo N, Ichihara MY, et al. Propensity score methods in health technology assessment: principles, extended applications, and recent advances. Front Pharmacol. 2019;10:973. 31619986 10.3389/fphar.2019.00973 PMC6760465

[16] Rubin DB. Using propensity scores to help design observational studies: application to the tobacco litigation. Health Serv Outcomes Res Methodol. 2001;2(3):169–88.

[17] Hoven-Gondrie ML, Bastiaannet E, Ho VK, van Leeuwen BL, Liefers GJ, Hoekstra HJ, et al. Worse survival in elderly patients with extremity soft-tissue sarcoma. Ann Surg Oncol. 2016;23(8):2577–85. 26957498 10.1245/s10434-016-5158-7 PMC4927613

[18] Al-Refaie WB, Habermann EB, Dudeja V, Vickers SM, Tuttle TM, Jensen EH, et al. Extremity soft tissue sarcoma care in the elderly: insights into the generalizability of NCI cancer trials. Ann Surg Oncol. 2010;17(7):1732–8. 20354801 10.1245/s10434-010-1034-z

[19] Casali PG, Abecassis N, Bauer S, Biagini R, Bielack S, Bonvalot S, et al. Soft tissue and visceral sarcomas: ESMO-EURACAN Clinical Practice Guidelines for diagnosis, treatment and follow-up. Ann Oncol. 2018;29(Supplement_4):iv51–67. 29846498 10.1093/annonc/mdy096

[20] Lindberg RD, Martin RG, Romsdahl MM, Barkley JHT. Conservative surgery and postoperative radiotherapy in 300 adults with soft-tissue sarcomas. Cancer. 1981;47(10):2391–7. 7272893 10.1002/1097-0142(19810515)47:10<2391::aid-cncr2820471012>3.0.co;2-b

[21] Leibel SA, Tranbaugh RF, Wara WM, Beckstead JH, Bovill JEG, Phillips TL. Soft tissue sarcomas of the extremities. Survival and patterns of failure with conservative surgery and postoperative irradiation compared to surgery alone. Cancer. 1982;50(6):1076–83. 7104949 10.1002/1097-0142(19820915)50:6<1076::aid-cncr2820500610>3.0.co;2-u

[22] Rosenberg SA, Tepper J, Glatstein E, Costa J, Baker A, Brennan M, et al. The treatment of soft-tissue sarcomas of the extremities: prospective randomized evaluations of (1) limb-sparing surgery plus radiation therapy compared with amputation and (2) the role of adjuvant chemotherapy. Ann Surg. 1982;196(3):305. 7114936 10.1097/00000658-198209000-00009 PMC1352604

[23] Beane JD, Yang JC, White D, Steinberg SM, Rosenberg SA, Rudloff U. Efficacy of adjuvant radiation therapy in the treatment of soft tissue sarcoma of the extremity: 20-year follow-up of a randomized prospective trial. Ann Surg Oncol. 2014;21(8):2484–9. 24756814 10.1245/s10434-014-3732-4 PMC6293463

[24] Pisters P, Harrison LB, Leung D, Woodruff JM, Casper ES, Brennan MF. Long-term results of a prospective randomized trial of adjuvant brachytherapy in soft tissue sarcoma. J Clin Oncol. 1996;14(3):859–68. 8622034 10.1200/JCO.1996.14.3.859

[25] Alektiar KM, Velasco J, Zelefsky MJ, Woodruff JM, Lewis JJ, Brennan MF. Adjuvant radiotherapy for margin-positive high-grade soft tissue sarcoma of the extremity. Int J Radiat Oncol Biol Phys. 2000;48(4):1051–8. 11072162 10.1016/s0360-3016(00)00753-7

[26] Kuklo TR, Temple HT, Owens BD, Juliano J, Islinger RB, Andejeski Y, et al. Preoperative versus postoperative radiation therapy for soft-tissue sarcomas. Am J Orthop (Belle Mead NJ). 2005;34(2):75–80. 15789525

[27] Larrier NA, Czito BG, Kirsch DG. Radiation therapy for soft tissue sarcoma: indications and controversies for neoadjuvant therapy, adjuvant therapy, intraoperative radiation therapy, and brachytherapy. Surg Oncol Clin N Am. 2016;25(4):841–60. 27591502 10.1016/j.soc.2016.05.012

[28] Koshy M, Rich SE, Mohiuddin MM. Improved survival with radiation therapy in high-grade soft tissue sarcomas of the extremities: a SEER analysis. Int J Radiat Oncol Biol Phys. 2010;77(1):203–9. 19679403 10.1016/j.ijrobp.2009.04.051 PMC3812813

