# Supplementary material for: High-risk soft-tissue sarcomas in elderly patients: does perioperative radiotherapy improve local control and prognosis?
Source: BMC Cancer. 2026 Jan 31;26:329. doi: 10.1186/s12885-026-15629-8 (PMC12977693; doi:10.1186/s12885-026-15629-8)
Supplement: Supplementary file 1 — Supplementary Material 1. [file 12885_2026_15629_MOESM1_ESM.pdf]

Supplementary Figure 1

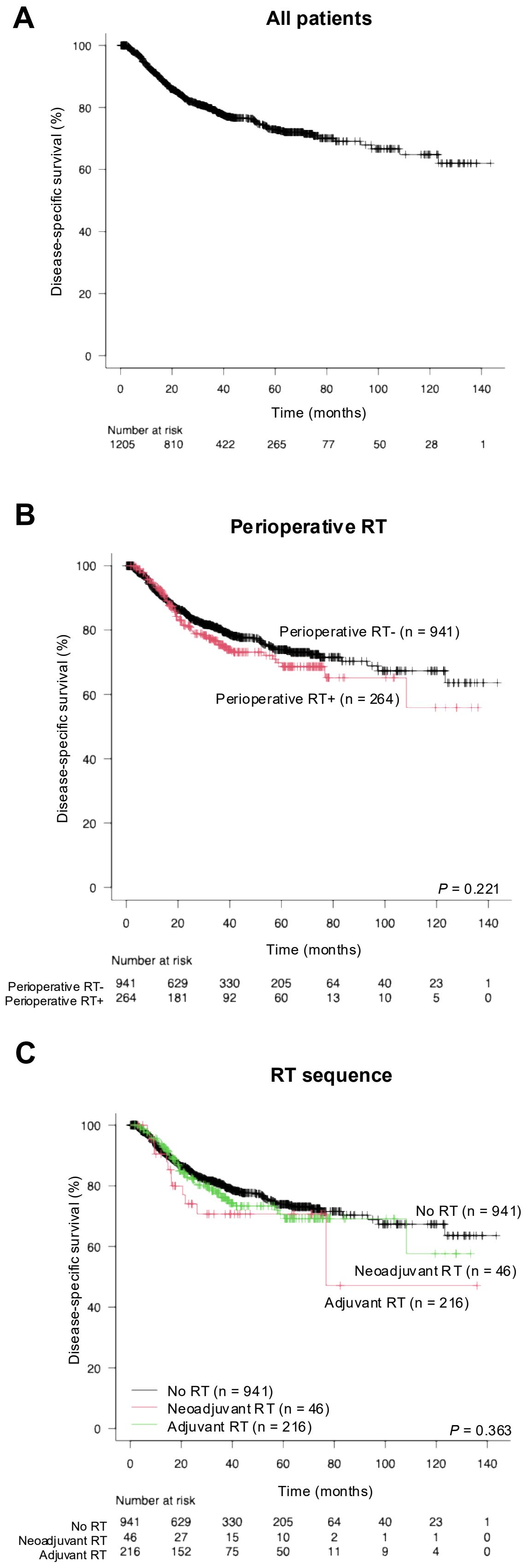

Supplementary Figure 1. (**A**) Kaplan-Meier curves showing the disease-specific survival in all patients. (**B,C**) Kaplan-Meier curves showing the disease-specific survival before propensity score matching, stratified by the administration of perioperative radiotherapy (RT) (*P* = 0.221; log-rank test) (**B**), and RT sequence (*P* = 0.363; log-rank test) (**C**).

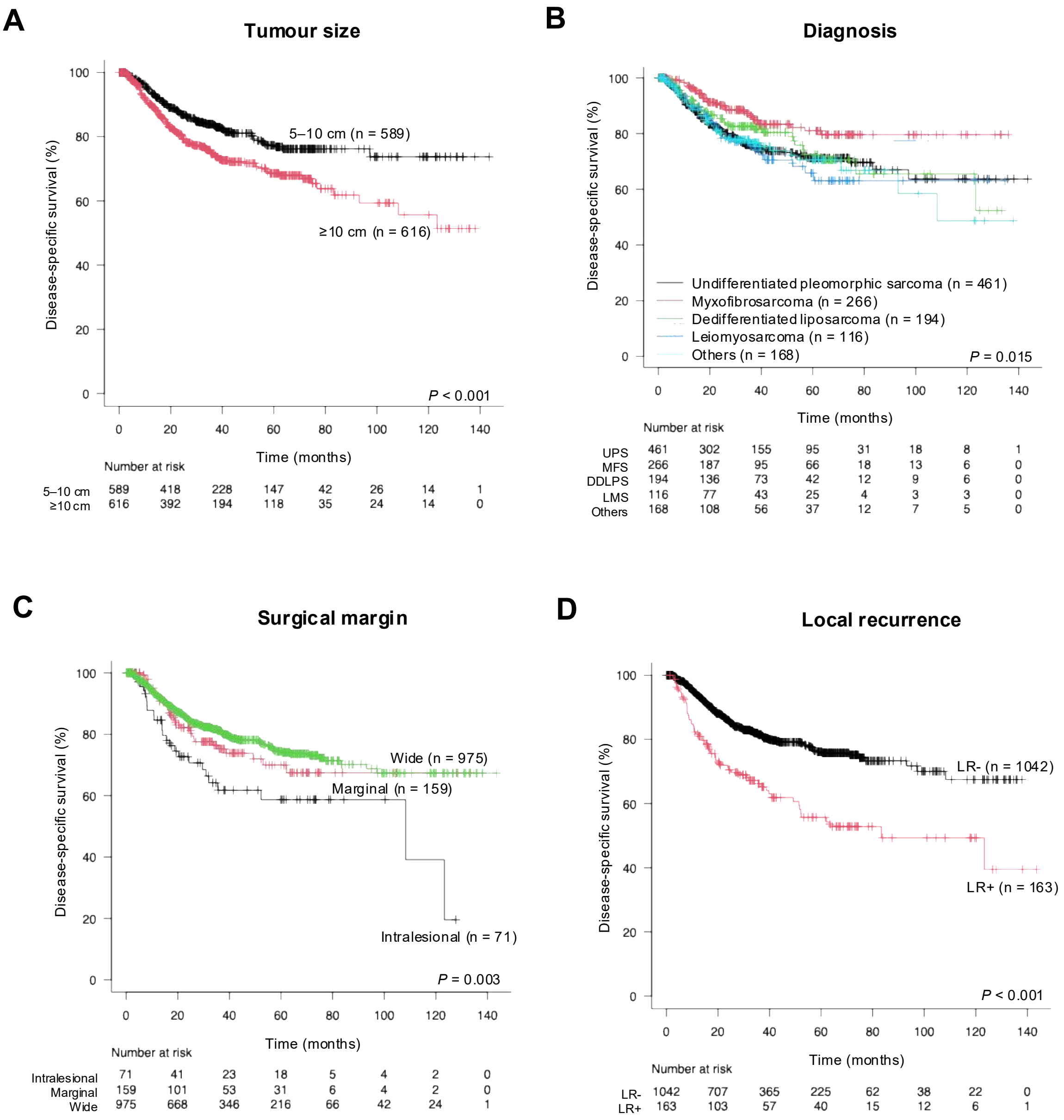

Supplementary Figure 2. Kaplan–Meier curves showing the disease-specific survival stratified by (**A**) tumour size (log-rank test, $P < 0.001$), (**B**) histological diagnosis (log-rank test, $P = 0.015$), (**C**) surgical margin (log-rank test, $P = 0.003$), and (**D**) local recurrence (log-rank test, $P < 0.001$).

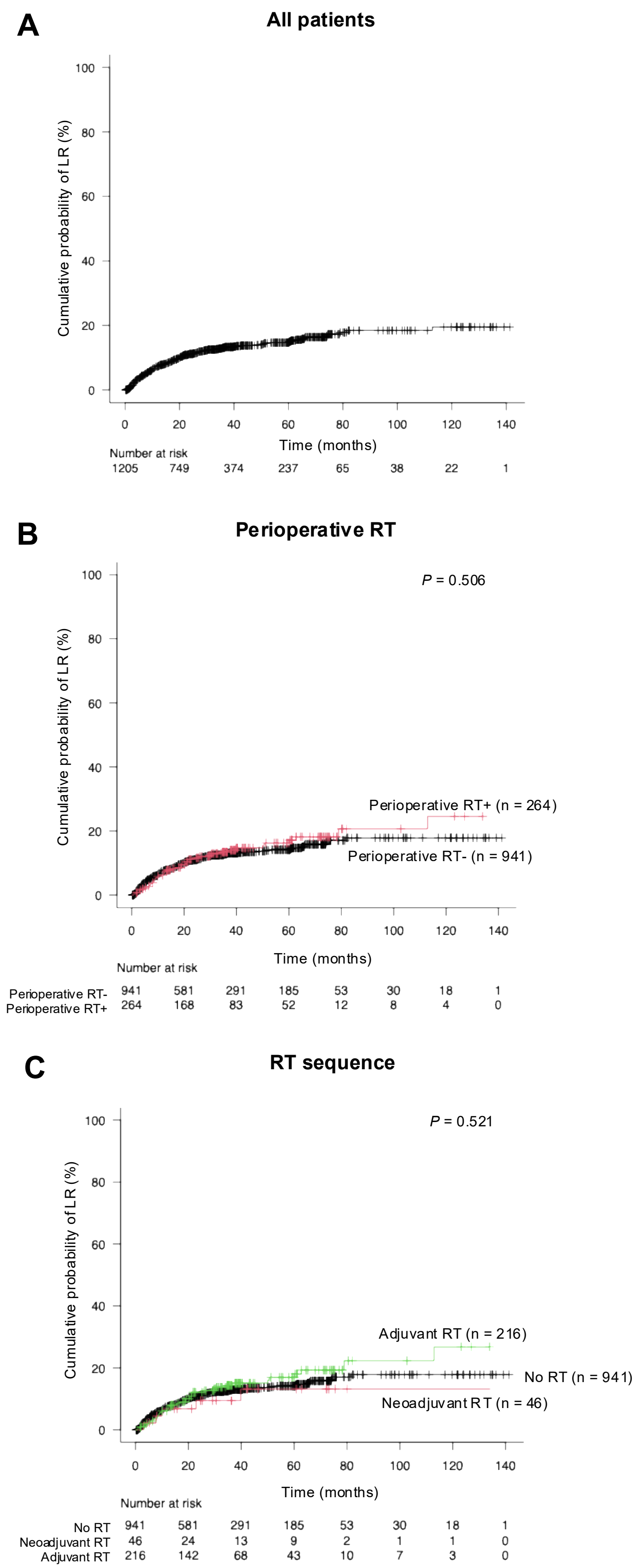

Supplementary Figure 3. (**A**) Cumulative probability of local recurrence in all patients. (**B,C**) Cumulative probability of local recurrence before propensity score matching, stratified by the administration of periopetative radiotherapy (RT) (*P* = 0.506; Gray's test) (**B**), and RT sequence (*P* = 0.521; Gray's test) (**C**).

Supplementary Figure 4

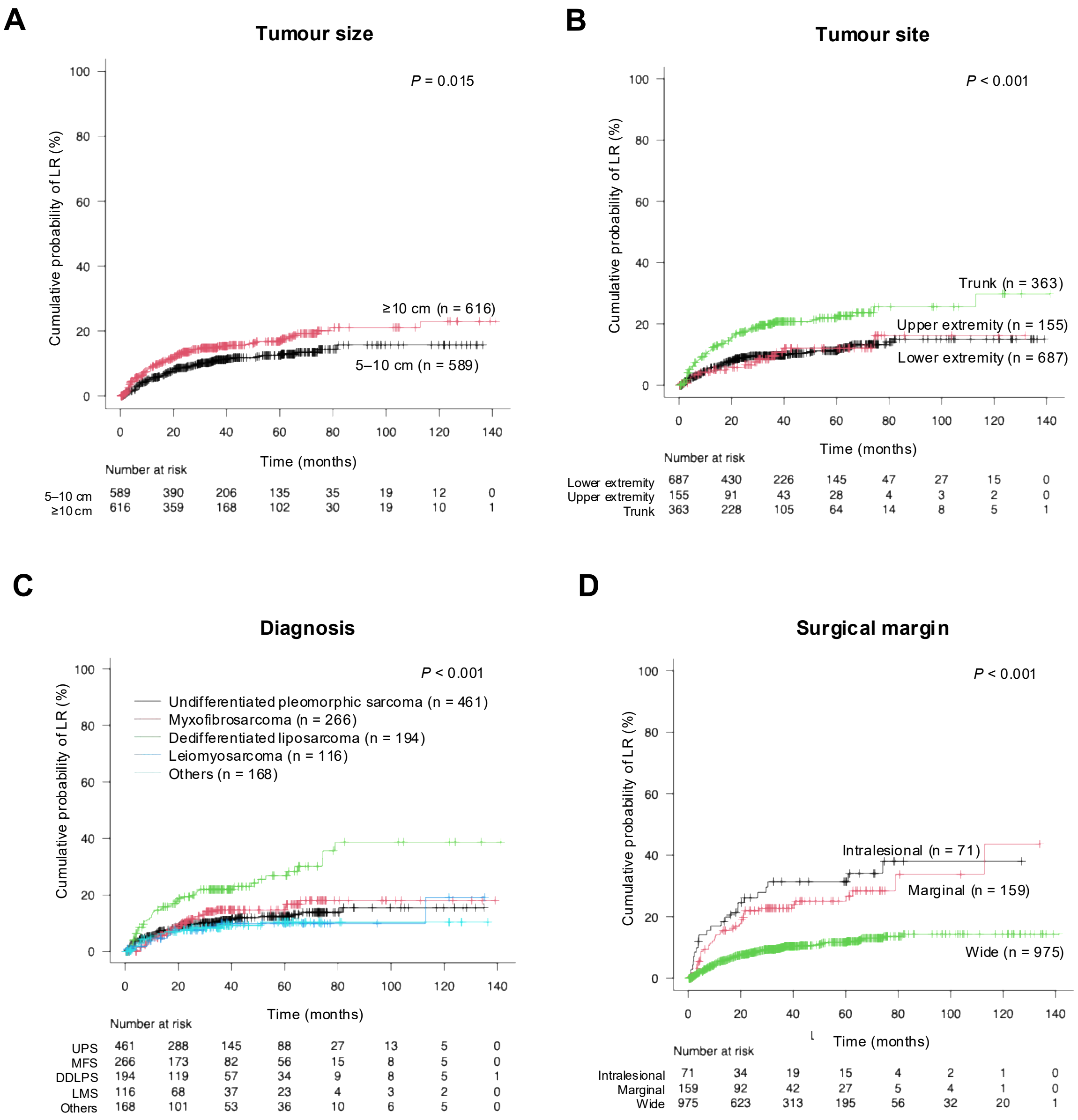

Supplementary Figure 4. Cumulative probability of local recurrence stratified by (**A**) tumour size (Gray's test, *P* = 0.015), (**B**) tumour site (Gray's test, *P* < 0.001), (**C**) histological diagnosis (Gray's test, *P* < 0.001), and (**D**) surgical margin (Gray's test, *P* < 0.001).

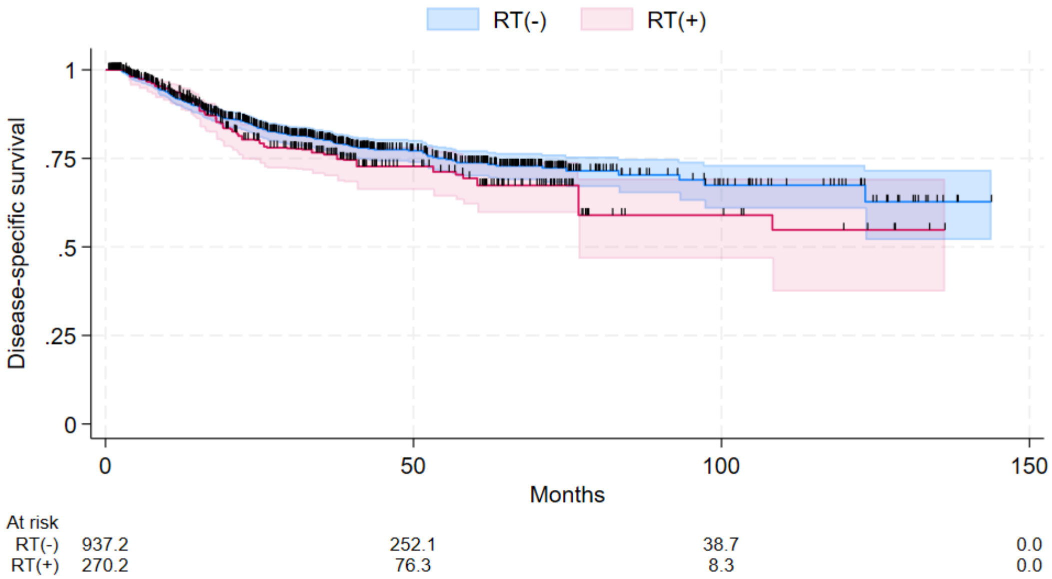

Supplementary Figure 5. Kaplan–Meier curve showing disease-specific survival (log-rank test, *P* = 0.130) after adjustment for potential confounders using stabilized inverse probability of treatment weighting (sIPTW).

Supplementary Table 1. Patient characteristics and their correlations with administration of perioperative RT

RT sequence	Total (n = 1205)		No (n = 941; 78%)		Yes (n = 264; 22%)		*P* value
	N	%	N	%	N	%	
Age							0.064
<80 years	688	57%	526	76%	162	24%	
≥80 years	517	43%	415	80%	102	20%	
Sex							0.112
Male	726	60%	576	79%	150	21%	
Female	479	40%	365	76%	114	24%	
Tumor site							0.001
Lower extremity	687	57%	552	80%	135	20%	
Upper extremity	155	13%	104	67%	51	33%	
Trunk	363	30%	285	79%	78	21%	
Size							0.005
<10 cm	589	49%	479	81%	110	19%	
≥10 cm	616	51%	462	75%	154	25%	
Diagnosis							0.061
UPS	461	38%	364	79%	97	21%	
Myxofibrosarcoma	266	22%	192	72%	74	28%	
Dedifferentiated LPS	194	16%	162	84%	32	16%	
Leiomyosarcoma	116	10%	91	78%	25	22%	
Others	168	14%	132	79%	36	21%	
Margin							<0.001
Intralesional	71	6%	34	48%	37	52%	
Marginal	159	13%	86	54%	73	46%	
Wide	975	81%	821	84%	154	16%	
LR							0.165
No	1042	86%	819	79%	223	21%	
Yes	163	14%	122	75%	41	25%	
Year							0.510
≤2010	235	20%	182	77%	53	23%	
2011-2015	410	34%	328	80%	82	20%	
2015-2020	560	46%	431	77%	129	23%	

Supplementary Table 2. Univariable and multivariable analyses for disease-specific survival

	Univariable			Multivariable		
	N	5-year DSS (%)	*P* value	HR	95% CI	*P* value
Age			0.177			
<80 years	688	74.5			Reference	
≥80 years	517	69.5		1.22	0.94–1.58	0.131
Sex			0.175			
Male	726	71.3				
Female	479	75.2				
Tumor site			0.607			
Lower extremity	687	73.8			Reference	
Upper extremity	155	73.2		0.87	0.56–1.34	0.531
Trunk	363	70.6		1.06	0.80–1.40	0.702
Size			<0.001			
<10 cm	589	77.3			Reference	
≥10 cm	616	68.7		1.53	1.17–1.99	0.002
Diagnosis			0.015			
UPS	461	71.2			Reference	
Myxofibrosarcoma	266	81.0		0.51	0.35–0.75	<0.001
Dedifferentiated LPS	194	72.5		0.67	0.45–0.98	0.038
Leiomyosarcoma	116	65.8		1.15	0.76–1.74	0.519
Others	168	70.5		1.07	0.74–1.56	0.705
Margin			0.003			
Wide	975	74.4			Reference	
Marginal	159	70.0		1.08	0.73–1.59	0.718
Intralesional	71	58.7		1.67	1.07–2.61	0.023
RT			0.221			
No	941	73.9			Reference	
Yes	264	69.8		1.07	0.78–1.46	0.680
LR			<0.001			
No	1042	76.1			Reference	
Yes	163	55.7		2.20	1.63–2.98	<0.001
Year			0.552			
≤2010	235	75.3				
2011-2015	410	71.0				
2015-2020	560	73.1				

Supplementary Table 3. Univariable and multivariable analyses for cumulative probability of local recurrence (Fine-Gray subdistribution hazard model)

	Univariable			Multivariable		
	N	5-year LR (%)	*P* value	HR	95% CI	*P* value
Age			0.848			
<80 years	688	14.8			Reference	
≥80 years	517	14.4		1.02	0.74–1.40	0.920
Sex			0.712			
Male	726	14.9				
Female	479	14.3				
Tumor site			<0.001			
Lower extremity	687	11.2			Reference	
Upper extremity	155	12.1		1.00	0.56–1.77	1.000
Trunk	363	21.9		1.82	1.32–2.51	<0.001
Size			0.015			
<10 cm	589	12.5			Reference	
≥10 cm	616	16.7		1.25	0.91–1.72	0.180
Diagnosis			<0.001			
UPS	461	12.4			Reference	
Myxofibrosarcoma	266	14.7		1.1	0.71–1.69	0.670
Dedifferentiated LPS	194	26.9		1.81	1.21–2.71	0.004
Leiomyosarcoma	116	9.9		0.90	0.47–1.72	0.750
Others	168	10.4		0.74	0.41–1.33	0.320
Margin			<0.001			
Wide	975	11.7			Reference	
Marginal	159	25.0		2.28	1.51–3.38	<0.001
Intralesional	71	31.4		3.27	1.02–5.28	<0.001
RT			0.506			
No	941	14.1			Reference	
Yes	264	16.3		0.86	0.58–1.27	0.440
Year			0.459			
≤2010	235	16.5				
2011-2015	410	13.3				
2015-2020	560	15.1				

Supplementary Table 4. Patient's background after propensity score matching

	Perioperative RT+		Perioperative RT-		*p* value
	N	%	N	%	
Age					0.848
<80 years	135	49%	138	51%	
≥80 years	93	51%	90	49%	
Sex					0.773
Male	138	49%	142	51%	
Female	90	51%	86	49%	
Tumour site					0.822
Lower extremity	123	51%	117	49%	
Upper extremity	35	47%	39	53%	
Trunk	70	49%	72	51%	
Size					0.127
<10 cm	90	43%	119	57%	
≥10 cm	138	56%	109	44%	
Diagnosis					0.298
UPS	81	47%	90	53%	
Myxofibrosarcoma	61	54%	53	46%	
Others	86	50%	85	50%	
Margin					0.753
Wide	152	50%	149	50%	
Marginal	55	51%	53	49%	
Intralesional	21	45%	26	55%	
Year					0.466
≤2010	45	56%	35	44%	
2011-2015	68	48%	73	52%	
2015-2020	115	49%	120	51%	

Supplementary Table 5. Covariate balance before and after propensity matching

	Before matching				After matching			
	RT(-) 941 (78.1%)	RT(+) 264 (21.9%)	SMD	VR	RT(-) 228 (50.0%)	RT(+) 228 (50.0%)	SMD	VR
Age (year)	78.95 (6.08)	78.33 (6.29)	-0.10	1.07	78.24 (6.27)	78.64 (6.45)	0.06	1.06
Sex								
Female	365 (38.8%)	114 (43.2%)	0.09	0.97	86.00 (37.7%)	90.00 (39.5%)	0.04	0.98
Male	576 (61.2%)	150 (56.8%)	-0.09	1.04	142.00 (62.3%)	138.00 (60.5%)	-0.04	1.02
Diagnosis								
UPS	364 (38.7%)	97 (36.7%)	-0.04	0.98	90.00 (39.5%)	81.00 (35.5%)	-0.08	0.96
MFS	192 (20.4%)	74 (28.0%)	0.18	1.25	53.00 (23.2%)	61.00 (26.8%)	0.08	1.10
Others	385 (40.9%)	93 (35.2%)	-0.12	0.95	85.00 (37.3%)	86.00 (37.7%)	0.01	1.00
Tumour site								
Lower extremity	552 (58.7%)	135 (51.1%)	-0.15	1.03	117.00 (51.3%)	123.00 (53.9%)	0.05	0.99
Upper extremity	104 (11.1%)	51 (19.3%)	0.23	1.59	39.00 (17.1%)	35.00 (15.4%)	-0.05	0.92
Others	285 (30.3%)	78 (29.5%)	-0.02	0.99	72.00 (31.6%)	70.00 (30.7%)	-0.02	0.98
Tumor size (cm)	11.43 (8.00)	11.64 (6.99)	0.03	0.76	11.27 (6.01)	11.85 (7.16)	0.09	1.42
Margin								
Wide	821 (87.2%)	154 (58.3%)	-0.69	2.19	149.00 (65.4%)	152.00 (66.7%)	0.03	0.98
Marginal	86 (9.1%)	73 (27.7%)	0.49	2.42	53.00 (23.2%)	55.00 (24.1%)	0.02	1.03
Intralesional	34 (3.6%)	37 (14.0%)	0.37	3.47	26.00 (11.4%)	21.00 (9.2%)	-0.07	0.83
Fiscal year category								
–2010	182 (19.3%)	53 (20.1%)	0.02	1.03	35.00 (15.4%)	45.00 (19.7%)	0.12	1.22
2011–2015	328 (34.9%)	82 (31.1%)	-0.08	0.95	73.00 (32.0%)	68.00 (29.8%)	-0.05	0.96
2016–2020	431 (45.8%)	129 (48.9%)	0.06	1.01	120.00 (52.6%)	115.00 (50.4%)	-0.04	1.00

SMD: Standardized mean diffrence.

VR: Variance ratio.

A SMD between –0.1 and +0.1, together with a VR within the range of 4/5 to 5/4, was considered acceptable to indicate covariate balance between the groups.

Supplementary Table 6. Covariate balance before and after propensity matching considering inverse probability of treatment weighting (IPTW)

	Before weighting				After weighting			
	RT(-) 941 (78.1%)	RT(+) 264 (21.9%)	SMD	VR	RT(-) 938 (77.6%)	RT(+) 270 (22.4%)	SMD	VR
Age (year)	78.95 (6.08)	78.33 (6.29)	-0.10	1.07	78.82 (6.14)	79.09 (6.56)	0.04	1.14
Sex								
Female	365 (38.8%)	114 (43.2%)	0.09	0.97	371.40 (39.6%)	102.17 (37.8%)	-0.04	1.01
Male	576 (61.2%)	150 (56.8%)	-0.09	1.04	566.68 (60.4%)	168.04 (62.2%)	0.04	0.99
Diagnosis								
UPS	364 (38.7%)	97 (36.7%)	-0.04	0.98	355.90 (37.9%)	98.00 (36.3%)	-0.03	0.98
MFS	192 (20.4%)	74 (28.0%)	0.18	1.25	209.78 (22.4%)	58.47 (21.6%)	-0.02	0.98
Others	385 (40.9%)	93 (35.2%)	-0.12	0.95	372.40 (39.7%)	113.74 (42.1%)	0.05	1.02
Tumour site								
Lower extremity	552 (58.7%)	135 (51.1%)	-0.15	1.03	531.99 (56.7%)	151.59 (56.1%)	-0.01	1.01
Upper extremity	104 (11.1%)	51 (19.3%)	0.23	1.59	122.04 (13.0%)	32.91 (12.2%)	-0.02	0.95
Others	285 (30.3%)	78 (29.5%)	-0.02	0.99	284.05 (30.3%)	85.70 (31.7%)	0.03	1.03
Tumor size (cm)	11.43 (8.00)	11.64 (6.99)	0.03	0.76	11.50 (7.90)	11.69 (6.05)	0.03	0.59
Margin								
Wide	821 (87.2%)	154 (58.3%)	-0.69	2.19	760.37 (81.1%)	221.49 (82.0%)	0.02	0.97
Marginal	86 (9.1%)	73 (27.7%)	0.49	2.42	123.15 (13.1%)	33.76 (12.5%)	-0.02	0.96
Intralesional	34 (3.6%)	37 (14.0%)	0.37	3.47	54.56 (5.8%)	14.96 (5.5%)	-0.01	0.96
Fiscal year category								
-2010	182 (19.3%)	53 (20.1%)	0.02	1.03	177.91 (19.0%)	47.02 (17.4%)	-0.04	0.94
2011-2015	328 (34.9%)	82 (31.1%)	-0.08	0.95	316.07 (33.7%)	93.09 (34.5%)	0.02	1.01
2016-2020	431 (45.8%)	129 (48.9%)	0.06	1.01	444.09 (47.3%)	130.10 (48.1%)	0.02	1.00

SMD: Standardized mean diffrence.

VR: Variance ratio.

A SMD between –0.1 and +0.1, together with a VR within the range of 4/5 to 5/4, was considered acceptable to indicate covariate balance between the groups.
